# Aberrant Resting-State Functional Connectivity in the Salience Network of Adolescent Chronic Fatigue Syndrome

**DOI:** 10.1371/journal.pone.0159351

**Published:** 2016-07-14

**Authors:** Laura Anne Wortinger, Tor Endestad, Annika Maria D. Melinder, Merete Glenne Øie, Andre Sevenius, Vegard Bruun Wyller

**Affiliations:** 1 Department of Pediatrics, Akershus University Hospital, Nordbyhagen, Norway; 2 Department of Psychology, University of Oslo, Oslo, Norway; 3 Cognitive Developmental Research Unit, Department of Psychology, University of Oslo, Oslo, Norway; 4 Research Department, Innlandet Hospital Trust, Lillehammer, Norway; Institute of Psychology, Chinese Academy of Sciences, CHINA

## Abstract

Neural network investigations are currently absent in adolescent chronic fatigue syndrome (CFS). In this study, we examine whether the core intrinsic connectivity networks (ICNs) are altered in adolescent CFS patients. Eighteen adolescent patients with CFS and 18 aged matched healthy adolescent control subjects underwent resting-state functional magnetic resonance imaging (*rf*MRI). Data was analyzed using dual-regression independent components analysis, which is a data-driven approach for the identification of independent brain networks. Intrinsic connectivity was evaluated in the default mode network (DMN), salience network (SN), and central executive network (CEN). Associations between network characteristics and symptoms of CFS were also explored. Adolescent CFS patients displayed a significant decrease in SN functional connectivity to the right posterior insula compared to healthy comparison participants, which was related to fatigue symptoms. Additionally, there was an association between pain intensity and SN functional connectivity to the left middle insula and caudate that differed between adolescent patients and healthy comparison participants. Our findings of insula dysfunction and its association with fatigue severity and pain intensity in adolescent CFS demonstrate an aberration of the salience network which might play a role in CFS pathophysiology.

## Introduction

Chronic fatigue syndrome (CFS) is characterized by unexplained, long-lasting, disabling fatigue and exertion intolerance, accompanied by pain, cognitive impairments, orthostatic problems and other symptoms [1]. CFS is a major cause of disability among adolescents and may have detrimental effects on psychosocial and academic development [2], as well as family functioning [3]. Adolescent CFS prevalence is estimated at 0.1% to 1.0% [4–6].

The pathophysiology of CFS remains poorly understood. Previous studies report enhanced sympathetic and attenuated parasympathetic cardiovascular nervous activity [7–9], low-grade systemic inflammation [10, 11], attenuation of the hypothalamus-pituitary-adrenal (HPA) axis [12, 13], and cognitive impairment of executive control functions [14, 15]. Recent evidence suggests that *hyperalgesia* is also a prominent feature of CFS [16, 17]. Winger and co-workers reported that adolescent CFS patients are characterized by increased pain severity scores and generalised lower pain pressure thresholds [16], pointing to a hypersensitivity phenomenon similar to fibromyalgia [18]. We have suggested that most of the documented features of CFS might be attributed to an *aberrant neurobiological stress response* or ‘*sustained arousal’* [19]. The neurobiological stress response is governed by highly automated brain networks involving autonomic, endocrine, and immune adjustments, and its initiation depends upon higher brain functions for the interpretation of sensory information and situations that threaten homeostasis [20].

Resting-state functional brain magnetic resonance imaging (*rf*MRI) is a powerful tool to characterize brain networks involved in normal brain activity as well as pathological states [21]. The triple network model (TNM) suggests that prominent features of several major psychiatric and neurological disorders might be related to aberration in three core intrinsic connectivity networks (ICN) of the brain, for review see: Menon (21). The ICNs are interdependent distributed networks that engage and disengage, often antagonistically, with general cognitive task demands. The three core ICNs are the default mode network (DMN), the central executive network (CEN), and the salience network (SN). The DMN is anchored in the posterior cingulate cortex and ventromedial prefrontal cortex (vmPFC). Alterations in DMN are associated with deficits in self-referential mental activity (i.e. rumination and hallucination) [22, 23]. The CEN encompasses dorsolateral prefrontal (dlPFC) and lateral posterior parietal cortices (PPC) and deficits in this network reflect impoverished cognition (i.e. working memory and executive control functions) [24–28]. The SN involves the cingulate-frontal operculum system whose integral hub is the anterior insula (AI). Weak interactions along the anterior–posterior axis of the insular cortex contribute to altered interoceptive awareness and monitoring of the internal milieu [29].

The sustained arousal model of CFS directly suggests alterations of the ICNs. Stress is related to alterations in facilitatory and inhibitory pathways and in large-scale functional neural networks of the central nervous system in both animal and human studies [30–34]. The well-documented cognitive dysfunction of adolescent CFS points to alterations in the CEN; whereas, the autonomic disturbances and augmented pain sensitivity might be related to alterations in the SN and particularly the insula, which plays a pivotal role in central autonomic control [35–37]. Accordingly, an adult CFS *rf*MRI analysis reported decreases in CEN and SN functional connectivity that correlated with fatigue symptoms [38]. Interestingly, associations between pain, insula, and DMN functional connectivity have been demonstrated in fibromyalgia [39], as well as changes in the intensity of intrinsic brain oscillations of the insula in irritable bowel syndrome [40], which are also phenotypically closely related to CFS.

The aim of this study was to compare the three core ICNs in adolescent CFS patients and healthy comparison participants. We hypothesized that intrinsic functional connectivity within the DMN, CEN and SN would be altered in adolescent CFS patients and that these alterations would be related to clinical symptoms of fatigue and pain.

## Method

This study is part of the NorCAPITAL-project (The Norwegian Study of Chronic Fatigue Syndrome in Adolescents: Pathophysiology and Intervention Trial) (Clinical Trials ID: NCT01040429). It was conducted at the Department of Pediatrics, Oslo University Hospital, Norway, which is a national referral center for young CFS patients. The current study is based on cross-sectional data collected during the first clinical in-hospital day of NorCAPITAL, from March 2010 to May 2012. All participants received a gift-card worth NOK 200. Informed, written consent was obtained from all participants and from parents/next-of-kin if required. The study was conducted in accordance with the Helsinki Declaration and approved by the Norwegian National Committee for Ethics in Medical Research.

### Participants

All hospital pediatric departments in Norway (n = 20), as well as primary care pediatricians and general practitioners, were invited to refer CFS patients aged 12–18 years consecutively to our department.

The referring units were equipped with written information for distribution to potential study participants and their parents/next-of-kin. If consent was given, a standard form required the referral unit to confirm the result of clinical investigations considered compulsory to diagnose pediatric CFS (pediatric specialist assessment, comprehensive hematology and biochemistry analyses, chest x-ray, abdominal ultrasound, and brain magnetic resonance imaging) [41]. Also, the referring units were required to confirm that the patient a) was unable to follow normal school routines due to fatigue; b) was not permanently bedridden; c) did not have any concurrent medical or psychiatric disorder that might explain the fatigue; d) did not experience any concurrent demanding life event (such as parents’ divorce) that might explain the fatigue; e) did not use pharmaceuticals (including hormone contraceptives) regularly. If medical history or current health status indicated a psychiatric condition, physicians were required to refer patients to a psychiatrist for evaluation. If a comorbid psychiatric disorder was found, those patients were removed from the study [42]. No patients received graded exercise therapy (GET) and two patients (out of the 18 viable resting-state MRI datasets) received cognitive behavioral therapy (CBT) at baseline. Completed forms were consecutively conveyed to the study center and carefully evaluated. Patients, considered eligible for this study, were summoned to a clinical meeting at our study center, and after which, a final inclusion decision was made.

In agreement with NICE clinical guidelines [41, 43], we applied a ‘broad’ case definition of CFS, requiring three months of unexplained, disabling chronic/relapsing fatigue of new onset. We did not require that patients meet any other accompanying symptom criteria, in contrast to the case definition from the International Chronic Fatigue Syndrome Study Group at the Centers for Disease Control and Prevention (commonly referred to as the Fukuda-definition), which appears to be most frequently used in the scientific community [44]. The Fukuda-definition requires at least six months of unexplained chronic or relapsing fatigue of new onset, severely affecting daily activities, as well as four or more of eight specific accompanying symptoms (headache, muscle pain, joint pain, sore throat, tender lymph nodes, impaired memory or concentration, unrefreshing sleep, and malaise after exertion). However, the validity of this definition has not been established [45]. In fact, several empirical findings raise concerns about the validity, in particular among adolescents: A formal factor analysis of symptoms in a broadly defined group of chronic fatigued patients did not show a strong correspondence with the Fukuda accompanying symptoms [46]. A study based upon the Swedish twin registry concluded that there was no empirical support for the requirement of four out of eight Fukuda accompanying symptoms [47]. A report on a broadly defined population of adolescent CFS patients concluded that the subgroup adhering to the Fukuda criteria was not characterized by a certain level of disability, nor was this subgroup specifically related to characteristics of underlying pathophysiology (alteration of cardiovascular autonomic control) [48]. Accordingly, subgrouping based upon the Fukuda criteria did not influence the cross-sectional comparisons or the intervention effects in previously reported results from the NorCAPITAL project [42]. Thus, the inclusion criteria in this study are wider than the Fukuda criteria. The main reason for not adhering to the Fukuda case definition was too few accompanying symptoms. Disease duration and percentage of patients fulfilling Fakuda and NICE criteria were reported.

In NorCAPITAL, a total of 120 CFS patients were included. This study is based upon a subset of patients generated from a computer-based randomization procedure, where one fourth of the patients were randomized to be included in the present study; 18 months disease duration served as stratification criterion [42]. The randomization procedure allocated 30 patients to fMRI assessment: of these, five patients did not want to participate in the study, four patients were excluded due to orthodontic treatment, two participants were removed due to scanning error, and one was excluded due to excessive movement > 3 mm in either of the three translation parameters or three rotation parameters, resulting in a total fMRI dataset of n = 18 adolescent CFS patients (mean age 15.9 years) for the final analyses. A group of 18 healthy controls (mean age 15.9 years) having a comparable distribution of gender and age were recruited from local schools. No chronic disease and no regular use of pharmaceuticals were allowed. All participants completed the Chalder Fatigue Questionnaire [49], Mood and Feelings Questionnaire for Depression [50], Spielberger State-Trait Anxiety Inventory [51], The Brief Pain Inventory (BPI) [52], and Wechsler Abbreviated Scale of Intelligence (WASI) [53]. Symptom data was missing at random for two of the patients, and the group mean was used for their lost data.

### Clinical Measures

The Chalder Fatigue Questionnaire [49], Spielberger State-Trait Anxiety Inventory [51], Mood and Feelings Questionnaire for Depression [50] were self-administered by all participants and returned in pre-stamped envelopes. The Brief Pain Inventory (BPI) [52] was completed prior to the fMRI scan.

The Chalder Fatigue Questionnaire is a valid outcome measure in adult [49] and adolescent CFS [54] based on symptoms during the preceding month. The sum across 11 items is scored on a 0–3 Likert scale, thus ranging from 0 (less severe fatigue) to 33 (more severe fatigue).

The Mood and Feelings Questionnaire (MFQ) has been validated in children and adolescents [50]. MFQ consists of 34 items to be rated based on symptoms during the preceding month. Each item is scored on a 0–2 Likert scale, and the total sum score is from 0 to 68. Higher scores imply more depressive symptoms.

The state anxiety measure from the Spielberger State-Trait Anxiety Inventory [51] is a valid measure of 12 items that asks participants to indicate how they feel right now on 4-point forced-choice Likert-type response scales. Scores range from 12 to 48, with higher scores suggesting greater levels of anxiety.

Brief Pain Inventory (BPI) assesses the intensities of pain and to what extent pain interferes with different aspects of life [52]. Each item from BPI was read aloud by one of the researchers and answered by the participant. Part 1 of the inventory contains single items of pain severity, of which a pain average item addresses pain during the last week and a pain now item addresses current pain. Both items are rated on 0–10 Likert scales, where higher scores signalize more severe pain.

### *rf*MRI Data Acquisition

Imaging data were collected on a 3T, Phillips Achieva whole-body scanner, with an 8 channel Philips SENSE head coil (Philips Medical Systems). Functional images were obtained with a single-shot T2*—weighted echo planar imaging sequence. Imaging sequence consisting of 250 volumes with: repetition time (TR): 2000 ms; echo time (TE): 30 ms; 3mm isotropic voxels; field of view (FOV): 240 x 240 reconstructed into 80x80; flip angle 80°; 38 transverse slices with 0 gap and scanned in a default interleaved sequence. The slices where collected starting from the bottom of the brain, collecting all the odd numbered slices first (1,3,5…) and then collecting all the even number slices (2,4,6..). The total scan time was 8 minutes. Participants were instructed to close their eyes and to rest comfortably, without moving or falling asleep, during the functional scan. For the 3D scan, an anatomical image with: TR: 10462 ms; TE: 54 ms; 2mm isotropic voxels; FOV: 224 x 224; flip angle 90°: 60 transverse slices with 0 gap and scanned in the default interleaved sequence.

### *rf*MRI Data Analysis

Analyses of the FMRI data were performed using the software package FSL (available from the FMRIB Software Library at www.fmrib.ox.ac.uk/fsl). Data were corrected for motion artifacts, compensated for any head movements using an FSL linear (affine) transformation (FSL-MCFLIRT) procedure. FSL brain extraction tool (FSL-BET) was used to skull strip the 3D T1 weighted image, and then used to skull strip functional data. The motion corrected and skull-stripped brain volumes were temporally high pass filtered (Gaussian-weighted LSF straight line subtraction, with σ = 100.0 s) and smoothed using a Gaussian kernel of 5-mm full width at half maximum. In-scanner motion parameters were calculated using frame displacement (FD) [55]. FD averages rotational and translational parameter differences, using weighted scaling, and was compared between groups using two-tailed independent samples *t*-test. Between group motion difference was considered significant at *P* < 0.05.

Analyses were performed using Independent Component Analysis (ICA) with the FSL Multivariate Exploratory Linear Optimized Decomposition into Independent Components (FSL-MELODIC) tool and a dual-regression approach [56, 57]. Functional data were first projected to standard Montreal Neurological Institute (2 mm MNI) space using structural/functional linear (affine) coregistration (FSL-FLIRT) and nonlinear structural/template coregistration using the FMRIB Nonlinear Image Registration Tool (FNIRT). These BOLD functional data (250 volumes for each subject) were then concatenated in time across all subjects, creating a single 4-dimensional (4-D) data set. We then applied probabilistic ICA to identify global and independent patterns of functional connectivity in the entire subject population, covering both CFS patients and healthy comparison group. We limited the number of independent components (ICs) in this step to 25. This was done to minimize IC splitting into subcomponents, as recommended by Filippini, MacIntosh (56).

From the 25 ICs, intrinsic connectivity networks (ICNs) of interest were selected on the basis of visual inspection from three independent experts. ICNs of interest were the DMN, CEN, and SN. Characteristically, the DMN includes the inferior parietal lobule (IPL) (Brodmann area (BA) 40, BA39), the posterior cingulate cortex (PCC) (BA30, BA23, BA31) and precuneus (BA7), areas of the medial frontal gyri (BA8, BA9, BA10, BA47), the hippocampal formation, and the lateral temporal cortex (BA21) [58]. The CEN is typically split, by ICA, into a right and a left lateralized network, and includes the dorsolateral prefrontal cortex (dlPFC), comprising (roughly) the frontal eye fields (BA4, BA6, BA8) and posterior parietal regions overlapping the superior parietal lobule (BA7) and intraparietal sulcus (iPS) (BA7, BA40) [59, 60]. SN includes dorsal anterior cingulate cortex (dACC) (BA 32), insular cortex (BA 13, 14) and two important subcortical structures, amygdala (BA 25) and substantia nigra/ventral tegmental area (SuN/VTA) [60].

The ICN spatial maps from the group-average analysis were used to generate subject-specific versions of the spatial maps and associated timeseries, using dual regression [56, 57]. For each subject, the group-average set of spatial maps was regressed (as spatial regressors in a multiple regression) into the subject's 4D space-time dataset. This resulted in a set of subject-specific timeseries, one per group-level spatial map. Next, those timeseries were regressed (as temporal regressors, again in a multiple regression) into the same 4D dataset, resulting in a set of subject-specific spatial maps, one per group-level spatial map. We then tested for group differences using FSL's randomize permutation-testing tool.

Group analyses were performed to evaluate differences in intrinsic brain connectivity between adolescent patients with CFS and healthy comparison group and to determine if this intrinsic connectivity was associated with symptom severity. Group main-effects maps for both CFS patients and comparison group, and between-group difference maps were determined for each of our ICNs of interest: the DMN, CEN, and SN. The FMRIB Local Analysis of Mixed Effects (FLAME) procedure was used, which involves Markov chain Monte Carlo sampling to estimate the true random-effects component of the between-subject mixed-effects variance (with degrees of freedom) at each voxel. For results from the difference map, clusters were determined by threshold-free cluster enhancement (TFCE) [61], corrected for multiple comparisons across voxels and for 3 ICNs of interest, and considered significant at *P* values less than 0.02. For significant ICN difference maps, post-hoc regression analysis was performed to access associations with fatigue symptom severity and extracted functional connectivity strengths between groups.

Secondly, we explored current pain intensity and pain average in two analyses of covariance to determine if group variance could be explained as a function of pain between SN group maps. Specifically, two groups with a continuous covariate interaction GLM was used for symptom scores of pain average and pain now, which were collected from the patients prior to the *rf*MRI scan. The ANCOVAs were performed using FLAME. Clusters were determined by TFCE [61], corrected for multiple comparisons across voxels and for 2 ANCOVAs, and considered significant at *P* values less than 0.03.

Functional connectivity Z scores, symptom measures and demographic data were evaluated using SPSS, version 22, (IBM Inc.; Chicago, IL), and between group differences were considered significant at *P* < 0.05.

## Results

Resting-state fMRI data were collected from 36 participants in the study. Adolescent CFS patient and comparison groups were well matched for age, gender, body mass index (BMI) and IQ; however, patients scored higher on clinical symptom scales (Table 1). As part of our dual-regression probabilistic ICA approach, we identified 25 ICs in the temporally concatenated 4D population data set, from which the DMN, CEN, and SN were robustly defined. The CEN was split into 2 lateralized networks, right CEN and left CEN.

**Table 1:** Demographic and clinical characteristics of adolescent Healthy Comparison Participants and Patients with Chronic Fatigue Syndrome.

Characteristic	Patients with Chronic Fatigue Syndrome (N = 18)		Healthy comparison group (N = 18)		p
	N	%	N	%	
Female	16	89	13	72	n.s.
^a^Fukuda criteria	13	81			
^b^NICE criteria	15	94			
	Mean	SD	Mean	SD	
Disease duration in months	19.1	9.8			
Age	15.9	1.5	15.9	1.6	n.s.
^c^BMI	22.8	3.4	20.6	2.7	n.s.
^d^WASI IQ	107.9	12.1	115.9	16.9	n.s.
Depression ^e^MFQ	16.1	7.8	6.7	7.7	<0.001*
Spielberger State Anxiety Inventory	21.1	4.8	16.0	3.9	0.001*
Chalder Fatigue Questionnaire	19.2	6.3	9.0	4.1	<0.001*
Pain Now ^f^BPI	2.5	2.0	0.8	1.8	0.013*
Pain Average ^g^BPI	5.0	2.2	2.8	1.7	0.002*
Motion during scanning					
Mean frame displacement^h^	0.11	0.04	0.13	0.06	n.s.

^a^Participants fulfilling the Fukuda-definition of CFS [44]

^b^Participants fulfilling the National Institute for Health and Care Excellence [43] definition of CFS

^c^Body Mass Index

^d^Wechlser Abbreviated Scale of Intelligence-estimated full IQ

^e^Mood and Feelings Questionnaire for Depression

^f^Pain now, rated prior to *f*MRI scan, item from the Brief Pain Inventory

^g^Pain average, rated for the past week, item from the Brief Pain Inventory

^h^Frame displacement [55]

*Indicates group comparison is significant at *p* < 0.05.

The χ^2^ test was used for sex; two-sample *t*-tests were used for continuous variables.

Not significant (n.s.)

**Fig 1:**
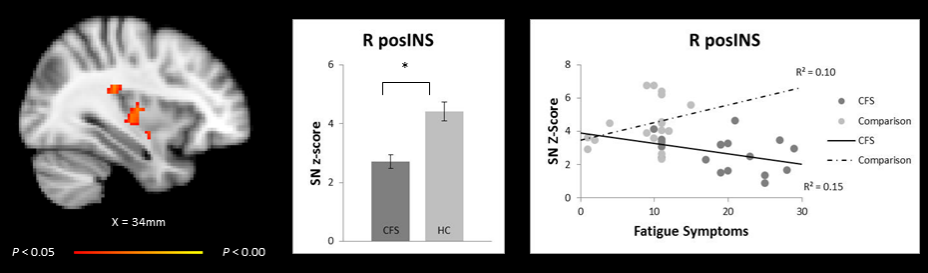
Salience network functional connectivity group difference map and its relationship with fatigue symptoms. Fig 1 difference map, subtracting adolescent CFS SN maps from healthy comparison (HC) SN maps, demonstrates less intrinsic SN connectivity to brain regions within the SN (right middle insula [R midINS], right posterior insula [R posINS], and right anterior insula [R antINS] **(left)**. Bar graph shows the mean and standard error Z scores for functional connectivity in each group **(middle)**. Right posterior insula connectivity strength decreases with greater fatigue symptoms in adolescent with Chronic Fatigue Syndrome but not with HC **(right)**. Dark circles represent individual patients with CFS and lighter circles represent HC participants. Activation height is thresholded at the *P* < 0.05 (corrected) level.

Of the ICNs evaluated, intrinsic connectivity within the SN demonstrated significant differences between patients with CFS and comparison participants. CFS patients exhibited decreased SN functional connectivity to the right insula: middle (midINS), posterior (posINS), and anterior (antINS) regions (Fig 1 and Table 2), and to brain regions outside of the classic boundaries of the SN, superior temporal gyrus, precentral gyrus and thalamus. No brain regions showed a greater correlation within the SN, DMN, and CEN in CFS patients as compared to healthy participants.

**Table 2:** Difference map for comparison of intrinsic connectivity between adolescent Healthy Comparison Participants and Patients with Chronic Fatigue Syndrome.

	Brain Area	side	# voxels	Between- group T score	Corrected *P* < value	Location			Z score by Group^¥^	
						x	y	z	Patients with CFS	Healthy Comparison
SN										
	Middle insula	R	17	3.93	0.03	34	-14	8	3.56 ± 3.59	4.78 ± 1.02
	Posterior insula	R	13	4.27	0.02*	30	-30	20	2.71 ± 1.02	4.41 ± 1.37
	Anterior insula	R	13	3.73	0.04	30	-10	-8	3.00 ± 1.27	4.09 ± 0.97
	STG	R	7	3.86	0.04	58	-26	8	3.81 ± 1.60	5.45 ± 1.54
	Precentral gyrus	L	5	4.25	0.04	-58	-2	20	1.97 ± 1.91	4.20 ± 1.08
	STG	R	4	3.71	0.04	58	-50	16	5.48 ± 1.62	7.54 ± 2.13
	Thalamus VLN	R	2	3.29	0.05	18	-14	4	0.68 ± 0.78	1.56 ± 0.93

CFS = Chronic Fatigue Syndrome; STG = superior temporal gyrus; VLN = Ventral Lateral Nucleus; R = right; L = left. Coordinates are given in MNI convention.

^¥^ Values are the mean ± SD within-group Z scores of intrinsic connectivity. Activation height is thresholded at the *P* < 0.05 (corrected) level.

*Significant at Bonferroni corrected level of *P* < 0.02.

In model one of the post-hoc regression, group and group*posINS interaction significantly predicted fatigue severity. In adolescent CFS patients, increases in fatigue symptoms were associated with decreases in SN functional connectivity to the right posINS; whereas in the healthy comparison group, increases in fatigue symptoms were related to increases in right posINS connectivity. The variables of model one accounted for 56% of the variance in fatigue scores. In model two, anxiety and depression variables did not influence the relationships of model one (Table 3 and Fig 1).

**Table 3:** Linear regression: adolescent CFS patients and comparison participant’s SN connectivity strengths to the right posterior insula associated with fatigue severity.

	Model 1			Model 2		
	*B*	*SE*	*ß*	*B*	*SE*	*ß*
**(constant)**	4.84	4.16		7.21	5.05	
**Group**	20.86	5.44	1.43**	19.14	5.97	1.32**
**posINS**	0.94	0.90	0.19	0.67	0.97	0.13
Group*posINS	-3.33	1.52	-0.70*	-3.25	1.53	-0.68*
**Depression**				0.18	0.14	0.22
**Anxiety**				-0.15	0.24	-0.10
***R***^***2***^		0.56			0.59	
***F***		13.65***			8.46***	
***Δ R***^***2***^					0.02	

Note

* *p* < 0.05

**p < 0.01

***p < 0.001

**Fig 2:**
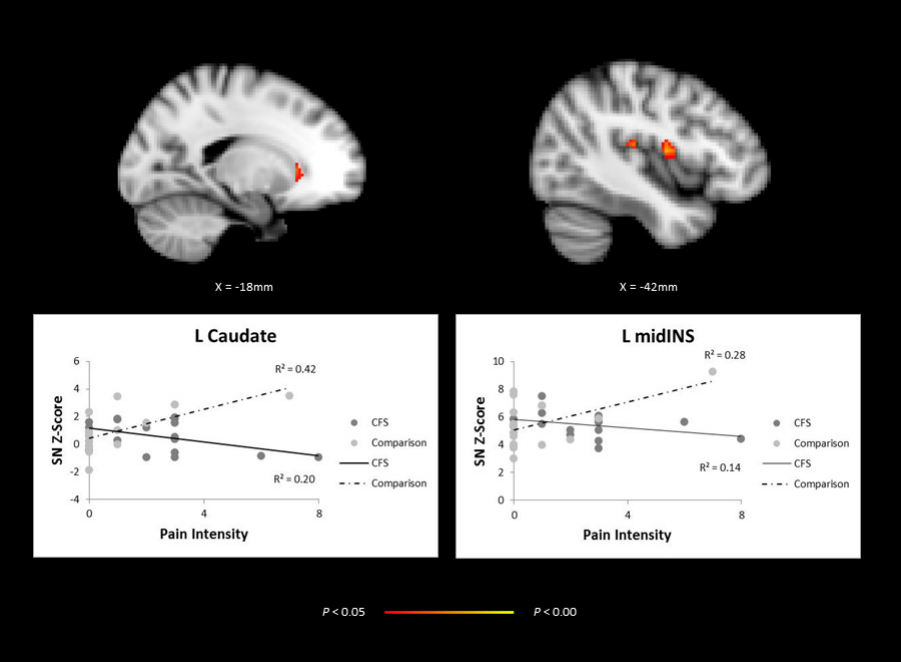
Covariation between salience network (SN) connectivity and pain intensity as assessed on functional magnetic resonance imaging. Fig 2 difference map, contrasting healthy comparison (HC) participants and adolescent patients with chronic fatigue syndrome (CFS), demonstrates a group interaction between pain intensity scores and intrinsic SN connectivity to the left (L) caudate and middle insula **(top).** The graphs contain connectivity Z scores and pain intensity scores, where dark circles represent individual patients with CFS and lighter circles represent HC participants **(bottom)**. Activation height is thresholded at the *P* < 0.05 (corrected) level.

For the analyses of covariance, we found a significant group interaction between pain intensity scores (Pain Now item from the BPI) and SN functional connectivity to the left middle and posterior insula and caudate nucleus (Table 4 and Fig 2). The linear relationship between pain intensities and connectivity strengths of the insula and caudate increased in healthy controls; whereas in patients, we did not find an increase in connectivity strengths with greater pain intensities. We did not find significant covariate interactions between groups on pain average symptom scores.

**Table 4:** Covariation between intrinsic salience network (SN) connectivity and pain intensity.

	Brain Area	side	# voxels	Between- group T score	Corrected*P* < value*	Location			Z score by Group^¥^	
						x	y	z	Patients with CFS	Healthy Comparison
SN										
	Middle insula	L	90	7.49	0.01	-50	2	0	5.46 ± 0.85	5.47 ± 1.67
	Posterior insula	L	30	5.76	0.03	-34	-26	16	6.61 ± 1.01	7.63 ± 1.78
	Caudate nucleus	L	5	7.26	0.03	-18	22	4	0.53 ± 1.11	0.88 ± 1.41

L = left. Coordinates are given in MNI convention.

^¥^ Values are the mean ± SD within-group Z scores of intrinsic connectivity where an interaction was observed with pain intensities (pain now item from the BPI rated on a 0–10 Likert scale; higher scores signalize more severe pain) at the time of the scan between patients with CFS and healthy comparison participants. Activation height is thresholded at the *P* < 0.05 (corrected) level.

*Significant at Bonferroni corrected level of *P* < 0.03.

We also tested for the influences of anxiety and depressive symptoms on SN group maps and found no significant group interactions.

## Discussion

The main findings of this study were the differences in intrinsic functional connectivity of the salience network (SN) among adolescent CFS patients and healthy comparison participants. A negative linear relationship with decreasing SN functional connectivity to the right insula and increasing fatigue symptoms was found in CFS patients, but this association was not observed in the healthy comparison participants. A negative linear relationship with decreasing SN functional connectivity to the left insula and caudate and increasing pain intensities was found in CFS patients, but this association was not observed in the healthy comparison participations.

Alterations of the SN in adolescent CFS are in-line with adult CFS resting-state functional connectivity studies [38, 62]; particularly, the SN functional connectivity decreases observed to the right insula in adult CFS patients [62]. Despite previous resting-state studies on adult CFS [62, 63], we did not find alterations in connectivity patterns within the DMN or CEN. However, using a similar approach as this study, a network connectivity analysis on adult CFS did not find alterations in DMN, either [38]. In contrast, they did find decreases within the CEN in adult CFS patients compared to the healthy group [38], as did [62]. The discrepancies in detecting DMN and CEN alterations in adolescent CFS are unclear and might be related to years of chronicity with the disease.

During adolescence, changes in the functional connectivity of brain networks are ongoing [64], and in particularly, alterations in adolescent DMN [65, 66]. There may be claims that the inconsistencies between DMN abnormalities in adult CFS and non DMN findings in adolescent CFS may be due to developmental differences. However, in many adolescent neuropsychiatric disorders, DMN connectivity abnormalities have been reported [67–69]. Nevertheless, the consistent decreases in SN functional connectivity found in previous adult CFS studies [38, 62] and in the adolescent CFS patients of this study, may implicate SN functional connectivity as having a role in the pathophysiology of CFS.

The SN plays a prominent role in the interactions with other brain networks: detecting and integrating salient sensory information [60, 70] and switching between DMN and CEN [71]. SN deviations seem to be common in disorders where there appears to be a disruption in the interpretation of salient biological and cognitively important information [72]. This atypical SN connectivity pattern to the insular cortex differentially increases or decreases in these disorders [39, 73–75]. Since the SN is a main player in brain network dynamics, it could be that SN alterations observed in this study might indicate a fundamental neural network change that is related to the onset of the CFS during adolescence.

The insular cortex is a complex structure for interoception, sense of the physiological condition of the body. Interoceptive signaling arrives in posterior insular regions [76] and is relayed to anterior regions [77], where interoceptive awareness has been extensively studied [77–80]. The lateralized relationship between SN functional connectivity decreases to the right posterior insula and subjective fatigue ratings in CFS is unclear. However, interoceptive awareness has been consistently reported with greater activation in the right anterior insula [36, 72]. Additionally, a decrease in right insula activity during a fatigue-inducing task was reported in adult CFS [81]. The imbalance in SN functional connectivity to the right posterior insula might be related to an abnormal assessment of interoceptive signaling (fatigue signaling in the body) along the right posterior to anterior insula axis that leads to heightened fatigue awareness in CFS.

The symptomatology of adolescent CFS is complex with a broad range of clinical symptoms and ailments [1]. A heightened sensitivity to external stimuli seems to be a problem across multiple sensory systems: light, sound, and pain, of adolescent CFS sufferers [42]. The association between SN decreases to the left middle insula and caudate and pain intensities in adolescent CFS patients indicates alterations in pain processing pathways [77, 82]. The pain intensity rating is a general feeling of bodily pain, so it is difficult to explain the left lateralization effect found in adolescent patients. Supporting this finding, a similar pattern of associations between alterations in functional connectivity to the left mid- and posterior insula and pain has been reported in other disorders [39, 73, 83]. The relationship between insula and caudate connectivity and pain intensities in adolescent CFS further implicates SN functional connectivity as having a role in the pathophysiology of CFS.

Within the adolescent CFS patients, psychiatric disorders were ruled out prior to inclusion in this study [42]. Even though they reported significantly more anxiety and depressive symptoms than the healthy comparison group, greater symptoms do not indicate the presence of an additional disorder. Of particular note, differences in intrinsic connectivity patterns of the SN in adolescent CFS patients of this study correlated more with their inability to inhibit fatigue and pain sensations than with other symptom comorbidities (e.g., depression or anxiety).

An aberrant neurobiological stress response, or *sustained arousal*, has been put forth as a model of disease mechanisms in CFS [19]. The identification of SN dysfunction in the adolescent CFS patients adds to the growing body of evidence of stress abnormalities in facilitatory and inhibitory neural pathways [31] and autonomic nervous system activity [48, 84]. Prolonged stress activation disrupts insular function and its ability to engage systems involved in the descending pain inhibition pathways [85, 86]. Although speculative, sustained arousal might explain the abnormal interaction of symptom severity and insula hypoconnectivity within the SN of adolescent CFS patients.

### Strengths and Limitation

Using an adolescent patient population, it might be easier to identify real disease mechanisms as opposed to secondary phenomena associated with years of chronicity. As in CFS, comorbid symptomatology (fatigue, pain, and depression) is apparent in many disorders, so future studies might want to systematically compare symptoms across adolescent disorders to better assess the specificity in neural changes.

Because of the common fluctuations in symptom severity in patients over time, it has been argued that a state measure, at the time of scanning, is on the same order as the low-frequency intrinsic connectivity of the ICNs [39], which might have less explanatory abilities when it comes to chronic alterations. Supporting this claim, a relationship between pain intensity, a measure of pain at the time of scanning, and SN functional connectivity was observed in the CFS patients of this study; however, the fatigue symptom scale that assesses fatigue during the preceding month was also related to SN functional connectivity in patients.

A small sample size might limit the generalizability of these results and increase the risk of type II errors. Furthermore, a causal relationship cannot be inferred in a cross-sectional design. We used liberal inclusion criteria, where not all patients adhere to the Fukuda-definition that is most widely accepted. Even so, we had a high dropout rate that might add to a selection bias.

The lack of DMN and CEN findings of this study might be due to the methodological approach, like regional versus network functional connectivity analyses. Future research might address seed-based approaches that restrict search volumes, so differences in functional connectivity might be easier to detect and identify much-needed biomarkers of CFS.

## Conclusion

Our findings of insula connectivity dysfunction and its association with somatic fatigue and pain in adolescent CFS demonstrate an aberration of the salience network, which might play a role in CFS pathophysiology. These findings may also have broader implications for how abnormalities in fatigue and pain perception arise from a complex interplay among brain networks and stress-related alterations in chronic fatigue syndrome.
